# Convergent Functional Changes of Default Mode Network in Mild Cognitive Impairment Using Activation Likelihood Estimation

**DOI:** 10.3389/fnagi.2021.708687

**Published:** 2021-10-05

**Authors:** Qianqian Yuan, Wenzhang Qi, Chen Xue, Honglin Ge, Guanjie Hu, Shanshan Chen, Wenwen Xu, Yu Song, XuLian Zhang, Chaoyong Xiao, Jiu Chen

**Affiliations:** ^1^Department of Radiology, The Affiliated Brain Hospital of Nanjing Medical University, Nanjing, China; ^2^Department of Neurosurgery, Nanjing Brain Hospital Affiliated to Nanjing Medical University, Nanjing, China; ^3^Department of Neurology, The Affiliated Brain Hospital of Nanjing Medical University, Nanjing, China; ^4^Institute of Neuropsychiatry, The Affiliated Brain Hospital of Nanjing Medical University, Fourth Clinical College of Nanjing Medical University, Nanjing, China

**Keywords:** mild cognitive impairment, default mode network, amplitude of low frequency fluctuation/fractional amplitude of low frequency fluctuation, regional homogeneity, functional connectivity

## Abstract

**Background:** Mild cognitive impairment (MCI) represents a transitional state between normal aging and dementia disorders, especially Alzheimer's disease (AD). The disruption of the default mode network (DMN) is often considered to be a potential biomarker for the progression from MCI to AD. The purpose of this study was to assess MRI-specific changes of DMN in MCI patients by elucidating the convergence of brain regions with abnormal DMN function.

**Methods:** We systematically searched PubMed, Ovid, and Web of science for relevant articles. We identified neuroimaging studies by using amplitude of low frequency fluctuation /fractional amplitude of low frequency fluctuation (ALFF/fALFF), regional homogeneity (ReHo), and functional connectivity (FC) in MCI patients. Based on the activation likelihood estimation (ALE) algorithm, we carried out connectivity modeling of coordination-based meta-analysis and functional meta-analysis.

**Results:** In total, this meta-analysis includes 39 articles on functional neuroimaging studies. Using computer software analysis, we discovered that DMN changes in patients with MCI mainly occur in bilateral inferior frontal lobe, right medial frontal lobe, left inferior parietal lobe, bilateral precuneus, bilateral temporal lobe, and parahippocampal gyrus (PHG).

**Conclusions:** Herein, we confirmed the presence of DMN-specific damage in MCI, which is helpful in revealing pathology of MCI and further explore mechanisms of conversion from MCI to AD. Therefore, we provide a new specific target and direction for delaying conversion from MCI to AD.

## Introduction

Mild cognitive impairment (MCI) is thought to be an intermediary stage between normal aging and AD (Wang et al., 2013; Giau et al., 2019). MCI patients present with memory impairment, but are able to maintain normal activities of daily living (Wang et al., 2018). To the best of our knowledge, one of the main AD symptoms is dysfunction of integrating semantic information into long-term effective memory, and this dysfunction is known to be correlated with MCI (Brueggen et al., 2016). It is important to note that AD pathology begins prior to clinical manifestations, and is irreversible (Lu et al., 2020). For this reason, carrying out a correct disease diagnosis and early intervention during MCI can effectively delay or even prevent occurrence of clinical dementia (Wang et al., 2015). In recent years, resting-state functional magnetic resonance imaging (rs-fMRI) has emerged as a powerful non-invasive to study internal brain functional connectivity as it does not require specific external tasks and is reliable (Wang et al., 2018). Therefore, our study utilized this approach to explore the presence of specific neuroimaging markers of DMN during MCI.

The human brain has many complex functional regions, separate and connected, that process different types of information (Horwitz, 2003). There are many networks in the human brain, including the DMN, the executive control network and the sensorimotor network. Several previous studies have summarized the brain regions of DMN (Yuan et al., 2016; Bi et al., 2018), which roughly includes the precuneus, posterior cingulate cortex (PCC), inferior parietal cortex, medial prefrontal cortex and hippocampus (Kang et al., 2018). Generally speaking, there are also abnormal changes that develop in large-scale network activity in AD. In the current functional neuroimaging research networks, DMN is known to be easily affected by the AD process (Weiler et al., 2014). Furthermore, activity in the DMN increased during quiet rest with eyes closed or simple visual fixations, while activity in the DMN continued to decrease when performing a variety of novel, attention-requiring, and non-self-referential tasks (Raichle, 2015). The discovery of the DMN has rekindled a long-standing interest in the importance of the brain's ongoing, or intrinsic, activity. Thus, we focused on DMN in this study.

As is known, AD is a disconnection syndrome that can be detected prior to the onset of cognitive impairment (Zhou et al., 2008), rather than just causing damage to isolated areas of the brain (Wang et al., 2018). AD affects the brain across many different levels, including molecular functions and neurotransmitter systems, which eventually leads to cognitive dysfunction (Kang et al., 2018). Currently, structural imaging markers, molecular imaging markers and functional imaging fractional markers can be useful diagnostic factors that play a role in the conversion of MCI to AD (Teipel et al., 2017). In recent years, fMRI has become increasingly important with regards to exploring neurological diseases of the brain, such as AD (Weiler et al., 2014). In AD patients, DMN is susceptible to neurodegeneration and is involved in early pathological changes (Wang et al., 2018). Aβ protein deposition and neurofibrillary tangles are both pathological characteristics of MCI that can further lead to degeneration of neuronal activity and functional impairment (Matura et al., 2020). According to the amyloid hypothesis, amyloid deposition is the main cause of AD (Matura et al., 2020). Several studies have suggested that Aβ42, total brain volume, and Tau protein are all predictors of conversion from MCI to AD, and that amyloid beta protein distribution overlaps with DMN in specific brain regions (Adriaanse et al., 2014). Furthermore, several studies suggest that DMN may be a biomarker for early prediction of AD. Currently, research of DMN has been applied to several diseases (Eyler et al., 2019). Therefore, it is reasonable to hypothesize that functional-specific changes in DMN can be utilized as evidence to support a diagnosis of MCI.

FC can often be seen as an indirect indicator of cross-synaptic activity, as it demonstrates connections between neural activity in different regions of the brain space (Balachandar et al., 2015). In this article, we focus on papers that evaluate connectivity through the correlation of BOLD (blood oxygenation level dependent) time series, although there are several other imaging methods that can evaluate functional connectivity. In MCI patients, FC decreased mainly in PCC, precuneus, and left PHG (Liu et al., 2012). Interestingly, reduction of PCC and precuneus is synchronous (Liu et al., 2012). A simultaneous increase of activity in some brain regions in MCI individuals is thought to compensate for defects in other areas (Farras-Permanyer et al., 2019). As far as we know, the ALFF technique has been shown to be reliable and useful in studying intrinsic or spontaneous brain activity among patients with MCI or AD (Zhen et al., 2018). The results of ALFF appearing in MCI are very interesting. Changes in the frontal lobes, temporal lobes, and parietal lobes are variable and can be either increased or decreased (Kang et al., 2018). Some have suggested that a decrease in ALFF in the temporal region may be the result of neurotangles, which is a claim that cannot be denied (Postema et al., 2019). ReHo is a reliable rs-fMRI analysis algorithm to explore local functional connectivity (Li et al., 2015). It has been shown that the ReHo of the left inferior parietal lobule, the medial prefrontal cortex, and the PCC/PCU are altered among patients with MCI (Zhang et al., 2012), and that in patients with AD, ReHo of some regions of the DMN decreases with disease severity (Wang et al., 2015). Changes of different indexes may represent different sensitivities, and the changes of multiple indexes in the same brain region at the same period can help improve sensitivity of diagnosis.

Hence, our aim was to comprehensively evaluate specific changes in DMN among patients with MCI. Additionally, we believe that three indicators of DMN will demonstrate special imaging anomaly markers. Similarly, we can have a deeper understanding of functional changes of DMN in MCI patients, further understand its pathological mechanism, and provide novel ideas to explore novel treatment directions.

## Method

### Search Strategy

We systematically and comprehensively searched PubMed, Ovid, and Web of science. The search terms were as follows: (1) “functional magnetic resonance imaging” OR “resting state” AND “mild cognitive impairment” AND “default mode network” OR “default network” AND “Functional connectivity.” (2) “functional magnetic resonance imaging” OR “resting state” AND “mild cognitive impairment” AND “regional homogeneity.” (3) “functional magnetic resonance imaging” OR “resting state” AND “mild cognitive impairment” AND “amplitude of low frequency fluctuation” OR “fractional amplitude of low frequency fluctuation.”

### Inclusion and Exclusion Criteria

Our entry criteria included (1) an original article published in a peer-reviewed journal. (2) Subjects were only recruited if they met the diagnostic criteria of MCI. (3) The study included an analysis of DMN in the resting state. (4) Results were obtained in standard stereotactic space using the Montreal Neurological Institute (MNI) or Talairach/Tournoux template. (5) The study was a cross-sectional and case-control design. When the study was a longitudinal study, we used baseline patient imaging data. Our exclusion criteria were as follows: (1) patients were diagnosed with cerebrovascular dementia, Lewy body's dementia, and other diseases such as Parkinson, (2) meta-analysis and review, (3) a lack of normal control group and coordinates, and (4) missing data in the literature.

### Data Extraction and Quality Assessment

Two researchers in our group extracted data from literature. First, we included patients with MCI. Second, we read each study to determine whether it had valid coordinates and outcomes, and whether it was a study of FC, ReHo, and ALFF/fALFF in the DMN. Finally, we extracted coordinates of the DMN in literature, transformed the T coordinates, and then worked with the method in the form of MNI coordinates. In case of disagreement between the two current researchers on the adoption of the article, a third researcher will vote on the decision. ALFF and fALFF were utilized to measure the amplitude of brain activity in spontaneous regions. ReHo has been shown to be highly reliable when studying the local consistency of the brain. FC is often used to indicate whether connections between brain regions have been disrupted or are compensatory. Hence, we divided the data into two groups based on the results of FC, ReHo and ALFF/fALFF (MCI > HC Group and MCI < HC Group), and then analyzed them using a computer computing software.

The ALE Algorithm used in this meta-analysis is available to the neuroimaging community in the form of the GINGERALE desktop application (http://brainmap.org/ale) (Zhang et al., 2012). ALE is a coordinate-based meta-analysis method that can reduce the bias of laboratory results. ALE does not consider the activation points in neuroimaging studies as single activation points, but rather as the spatial probability distribution with given coordinates as the center, and then calculates the activation probability of each coordinate and draws an ALE map (Zhang et al., 2012). It has been widely utilized in rs-fMRI studies (Eickhoff et al., 2012).

### Data Analysis Procedures

First, we divided subjects into the normal control group and MCI patient group. Then, three indexes (ALFF, FC, ReHo) were compared between the two groups. Finally, results from the comparison of the three indicators were divided into two groups: the ascending group and the descending group. The results of the two groups are also discussed. We utilized a software to calculate increased ALFF/fALFF (*n* = 75; foci14), decreased ALFF/fALFF (*n* = 99; foci16), increased ReHo (*n* = 148; foci18), decreased ReHo (*n* = 202; foci25), increased FC (*n* = 604; foci85), and decreased FC (*n* = 388; foci169).

## Results

### Search Results

**Figure 1:**
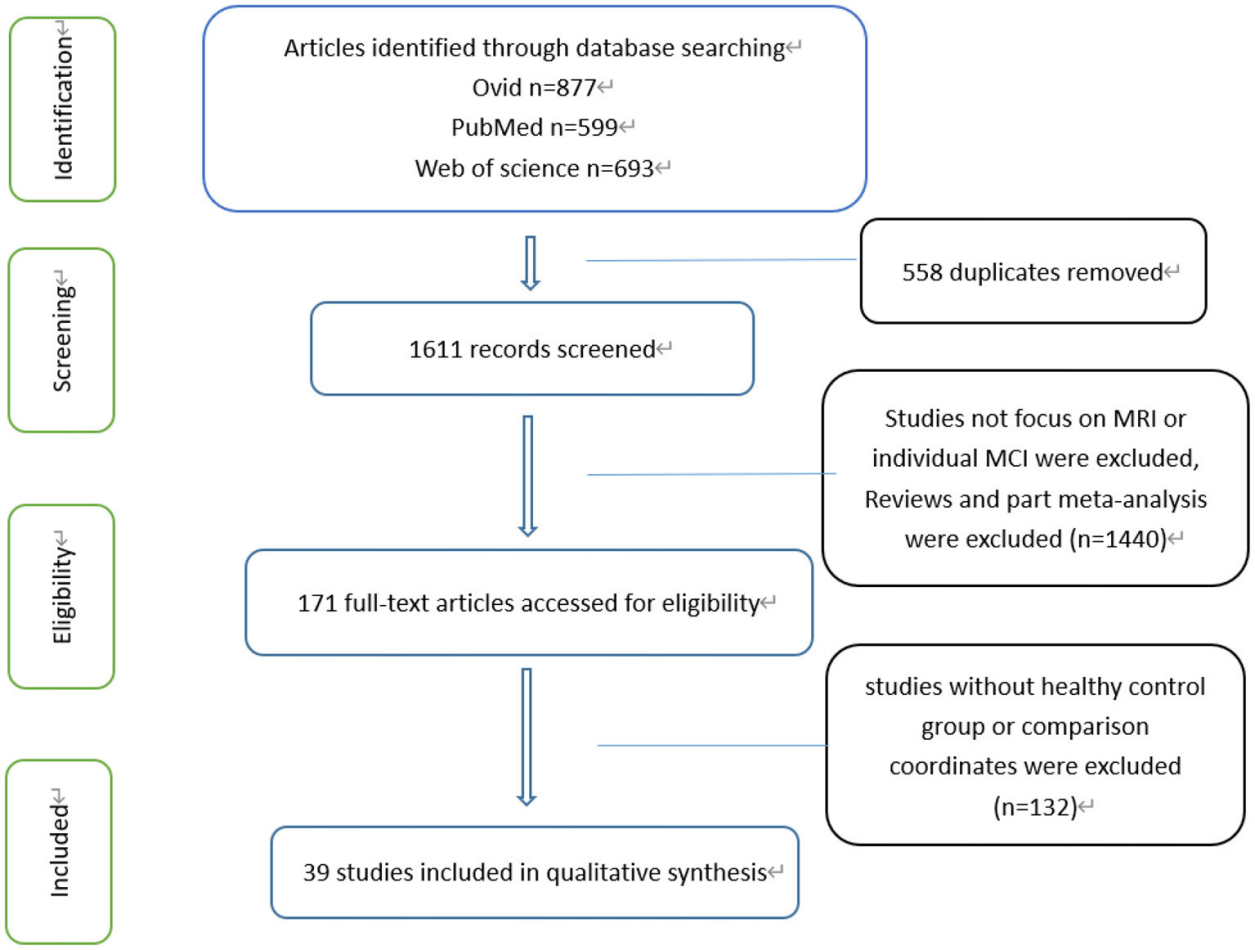
Flow of information through different phases of a systematical review.

Overall, 1,839 articles were identified across three different databases, from which 511 were duplicated and 1,328 were removed. In total, 39 articles were included, all of which included comparisons between the MCI patient group and a normal control group. Each of the 39 articles has complete data coordinates and meaningful results (Figure 1). In addition, there were 27 references included in FC, 27 references with declining FC and 17 references with rising FC. Fifteen references had both rising and declining results. There were seven references included in ReHo, seven references with declining ReHo and five references with rising ReHo. Five references had both rising and declining results. There were five references included in ALFF, three references with a decline in ALFF and five references with rise in ALFF, among which three had both rising and declining results. This meta-analysis incorporates literature and summary of relevant information as shown in Table 1.

**Table 1:** Demographic characteristics of the included studies.

**References**	***N***	**Age (SD)**	**Gender (M/F)**	**MMSE (SD)**	**Group contrasts**	**Foci**
**FC**						
Cai et al. (2015)	MCI 55	73.385 (7.61)	29/26	26.895 (1.73)	MCI>HC	0
	HC 30	75.8 (7.14)	13/17	29.63 (1.52)	MCI < HC	20
Joo et al. (2017)	MCI 50	72.1 (3.8)	23/27	24.4 (3.3)	MCI>HC	19
	HC 50	71.2 (4.3)	22/28	28.4 (1.5)	MCI < HC	3
Su et al. (2017)	MCI 80	69.9 (7.3)	38/42	26.2 (1.7)	MCI>HC	3
	HC 127	68.3 (6.6)	64/63	28.6 (1.4)	MCI < HC	5
Li et al. (2017)	MCI 25	64.56 (4.98)	9/16	26.88 (1.856)	MCI>HC	2
	HC 25	62.84 (2.79)	11/14	27.60 (1.732)	MCI < HC	1
Balachandar et al. (2015)	MCI 15	67.33 (6.6)	6/9	/	MCI>HC	2
	HC 15	64.4 (8.9)	6/9	/	MCI < HC	3
Wang et al. (2011)	MCI 14	69.64 (6.88)	8/6	26.64 (1.01)	MCI>HC	5
	HC 14	68.07 (7.46)	8/6	28.57 (0.65)	MCI < HC	28
Yi et al. (2015)	MCI 20	70.95 (2.105)	2/8	23.55 (0.83)	MCI>HC	5
	HC 12	71.75 (1.21)	3/9	27.40 (0.45)	MCI < HC	1
Han et al. (2012)	MCI 40	86.26 (4.49)	33/7	27.10 (1.96)	MCI>HC	4
	HC 40	86.28 (4.39)	25/15	28.68 (1.23)	MCI < HC	8
Lee et al. (2016)	MCI 87	71.4 (7.45)	43/44	27.85 (1.7)	MCI>HC	0
	HC 43	74.5 (5.8)	18/25	28.7 (1.4)	MCI < HC	19
Li et al. (2020)	MCI 30	68.53 (2.97)	13/17	25.10 (0.66)	MCI>HC	0
	HC 30	68.67 (3.19)	14/16	28.20 (0.92)	MCI < HC	1
Xue et al. (2019)	MCI 48	64.95 (8.57)	17/21	27.575 (1.8685)	MCI>HC	5
	HC 21	57.52 (8.07)	7/14	28.81 (1.209)	MCI < HC	0
Wang et al. (2015)	MCI 18	73.7 (9.1)	8/10	27.9 (1.2)	MCI>HC	4
	HC 16	70.7 (6.0)	4/12	/	MCI < HC	0
Barban et al. (2017)	MCI 23	70.45 (6.2)	14/9	/	MCI>HC	3
	HC 25	72.1 (6.15)	7/18	/	MCI < HC	0
Bharath et al. (2017)	MCI 48	67.22 (8.00)	13/35	/	MCI>HC	0
	HC 48	65.89 (7.20)	13/35	/	MCI < HC	1
Agosta et al. (2012)	MCI 12	69.1 (7.4)	6/6	26 (1)	MCI>HC	0
	HC 13	68.5 (6.9)	5/8	29 (1)	MCI < HC	1
Cha et al. (2013)	MCI 34	68.4 (7.9)	18/16	27.1 (2.1)	MCI>HC	0
	HC 62	68.5 (8.0)	17/45	28.6 (1.9)	MCI < HC	3
De Vogelaere et al. (2012)	MCI 16	67.2 (7.9)	8/8	24.4 (3.1)	MCI>HC	7
	HC 16	62.1 (6.8)	10/6	28.6 (1.3)	MCI < HC	16
Qi et al. (2010)	MCI 14	71.8 (7.3)	6/8	26.6 (0.3)	MCI>HC	5
	HC 14	70.4 (5.8)	8/6	28.5 (0.2)	MCI < HC	7
Bai et al. (2009)	MCI 30	72.5 (4.4)	15/15	27.0 (1.5)	MCI>HC	5
	HC 26	71.6 (5.3)	12/14	28.2 (1.4)	MCI < HC	0
Krajcovicova et al. (2017)	MCI 17	73.56 (6.64)	11/6	26.94 (1.68)	MCI>HC	0
	HC 18	68.22 (8.78)	5/13	29.17 (0.71)	MCI < HC	1
Bosch et al. (2010)	MCI 15	74.63 (6.85)	6/9	25.50 (2.03)	MCI>HC	3
	HC 15	72.20 (5.75)	5/10	27.67 (1.49)	MCI < HC	3
Yao et al. (2014)	MCI 13	75.5 (8.7)	8/5	25.8 (3.4)	MCI>HC	1
	HC 13	74.5 (8.7)	8/5	27.3 (1.8)	MCI < HC	5
Conwell et al. (2018)	MCI 15	71.1 (6.0)	9/6	25.0 (3.4)	MCI>HC	0
	HC 15	67.3 (8.4)	9/6	29.5 (0.6)	MCI < HC	1
Gardini et al. (2015)	MCI 21	70.62 (4.66)	13/8	/	MCI>HC	3
	HC 21	69.75 (6.45)	7/14	/	MCI < HC	0
Wang et al. (2011)	MCI 14	69.64 (6.88)	8/6	26.64 (1.01)	MCI>HC	0
	HC 14	68.07 (7.46)	8/6	28.57 (0.65)	MCI < HC	20
Li et al. (2016)	MCI 14	67.9 (9.5)	14/17	23.5 (2.9)	MCI>HC	0
	HC 14	65.6 (7.1)	15/27	28.0 (2.3)	MCI < HC	9
Yan et al. (2013)	MCI 18	66.7 (8.9)	11/7	24.3 (1.5)	MCI>HC	9
	HC 18	64.9 (8.4)	10/8	29.5 (0.5)	MCI < HC	7
**ALFF/fALFF**						
Yin et al. (2014)	MCI 11	66.6 (8.7)	2/9	24.6 (3.2)	MCI>HC	2
	HC 22	62.1 (8.1)	12/10	29.2 (1.1)	MCI < HC	4
Wang et al. (2011)	MCI 16	69.38 (7.00)	7/9	26.50 (1.03)	MCI>HC	8
	HC 22	66.55 (7.67)	7/15	28.59 (0.59)	MCI < HC	10
Cai et al. (2015)	MCI 39	72.4 (5.01)	20/19	25.51 (2.88)	MCI>HC	2
	HC 38	73.92 (3.90)	19/19	29.28 (0.88)	MCI < HC	3
Jing et al. (2012)	MCI 10	75.35 (6.45)	5/5	/	MCI>HC	4
	HC 8	78.42 (9.65)	3/5	/	MCI < HC	0
Zhao et al. (2015)	MCI 34	68.0 (7.6)	14/20	25.5 (1.6)	MCI>HC	0
	HC 34	66.9 (6.7)	18/16	29.2 (0.9)	MCI < HC	2
**ReHo**						
Cai et al. (2015)	MCI 102	72.015 (6.475)	52/50	24.27 (2.7)	MCI>HC	2
	HC 53	76.08 (6.45)	29/24	28.2 (2.13)	MCI < HC	2
Luo et al. (2018)	MCI 64	73.665 (4.76)	34/30	27.75 (1.695)	MCI>HC	2
	HC 49	73.33 (4.60)	18/31	29.02 (1.20)	MCI < HC	2
Wang et al. (2015)	MCI 32	69.1 (5.8)	18/12	26.2 (2.2)	MCI>HC	6
	HC 30	70.1 (5.5)	15/17	28.1 (1.5)	MCI < HC	5
Bai et al. (2008)	MCI 20	71.3 (3.8)	10/10	28.3 (1.4)	MCI>HC	1
	HC 20	69.4 (3.8)	9/11	27.2 (1.6)	MCI < HC	5
Kang et al. (2018)	MCI 34	76.1 (4.7)	7/27	22.7 (3.9)	MCI>HC	0
	HC 38	74.0 (5.4)	19/19	26.3 (2.3)	MCI < HC	2
Min et al. (2019)	MCI 10	69.80 (2.658)	5/5	25.90 (0.738)	MCI>HC	3
	HC 10	69.90 (2.601)	5/5	29.30 (0.823)	MCI < HC	5
Yuan et al. (2016)	MCI 36	66.8 (9.5)	17/19	24.9 (3.4)	MCI>HC	6
	HC 46	64.3 (7.8)	19/27	28.5 (2.0)	MCI < HC	8

### Meta-Analysis Results

#### Altered ALFF/fALFF in MCI

**Figure 2:**
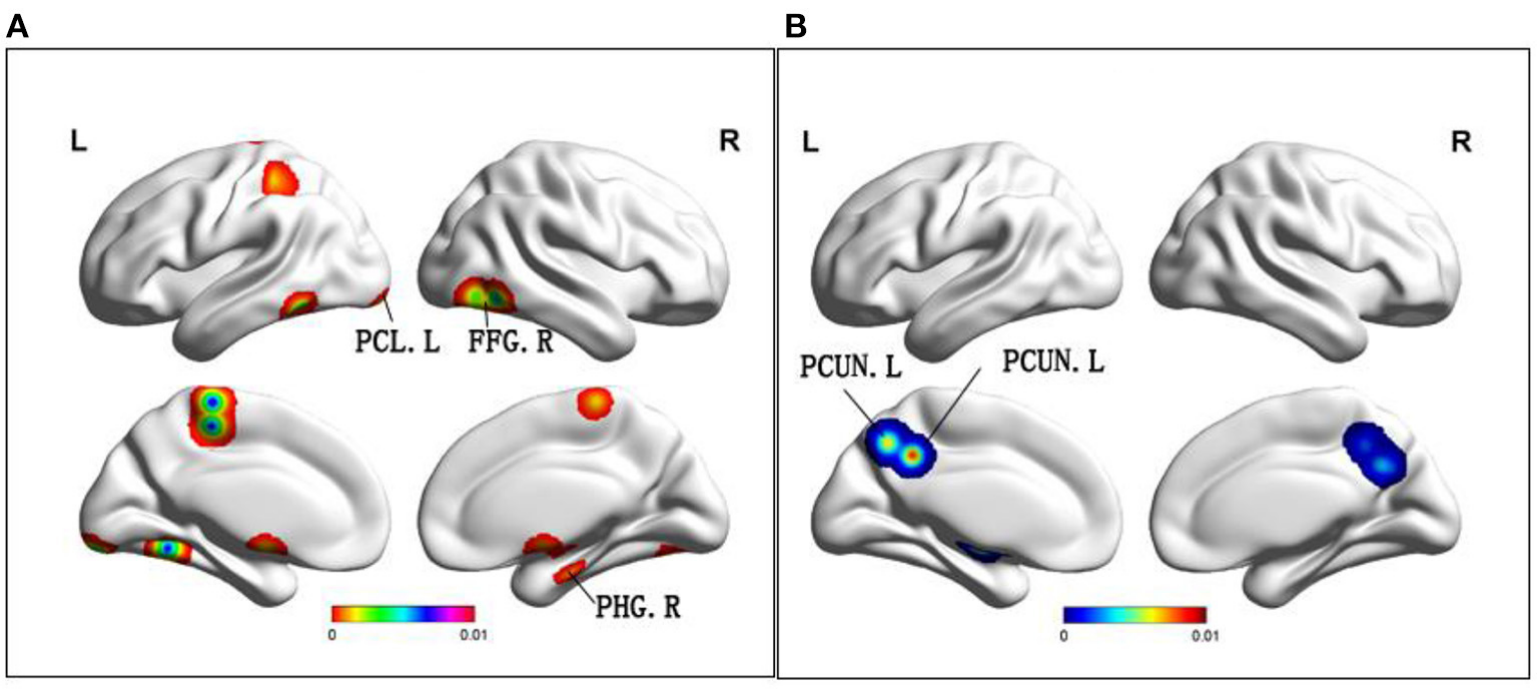
**(A)** Brain regions showing increased ALFF in MCI patients compared to HCs. **(B)** Brain regions showing decreased ALFF in MCI patients compared to HCs. MCI, amnestic mild cognitive impairment; HCs, healthy controls; ALFF, the amplitude of low frequency fluctuation; PCL, paracentral lobule; FFG, fusiform gyrus; PHG, parahippocampal gyrus; PCUN, precuneus; R, right; L, left.

Compared to HC, MCI patients demonstrated increased ALFF/fALFF in the bilateral cerebellum posterior lobe, right parahippocampal gyrus and bilateral fusiform gyrus (Table 2 and Figure 2). In addition, MCI patients had decreased ALFF/fALFF in the bilateral precuneus (Table 2 and Figure 2).

**Table 2:** All clusters from ALE analysis.

**Cluster**	**Volume (mm^**3**^)**	**MNI**			**Anatomical regions**	**Maximum ALE value**	**Side**	**BA**
		**X**	**Y**	**Z**				
**FC**								
MCI>HC								
1	848	4	58	−14	Medial Frontal Gyrus	0.015601669	Right	10
1	848	−6	56	−12	Anterior Cingulate	0.01257849	Left	10
**MCI < HC**								
1	9,168	2	−68	56	Precuneus	0.009993	Left	7
1	9,168	0	−66	50	Precuneus	0.009981	Left	7
1	9,168	12	−54	48	Precuneus	0.009978	Right	7
1	9,168	−4	−62	38	Precuneus	0.009745	Left	7
1	9,168	12	−68	60	Superior Parietal Lobule	0.009298	Right	7
1	9,168	0	−80	42	Cuneus	0.008475	Left	19
1	9,168	4	−56	40	Precuneus	0.008349	Right	7
1	9,168	2	−72	42	Precuneus	0.008181	Left	7
1	9,168	0	−46	40	Precuneus	0.00759	Left	31
1	9,168	10	−42	48	Cingulate Gyrus	0.007052975	Right	31
2	6,776	−46	−74	8	Middle Occipital Gyrus	0.015483191	Left	19
2	6,776	−56	−62	24	Superior Temporal Gyrus	0.011498914	Left	39
2	6,776	−54	−64	28	Middle Temporal Gyrus	0.010231528	Left	39
2	6,776	−54	−58	36	Superior Temporal Gyrus	0.0014	Left	39
2	6,776	−36	−78	24	Middle Temporal Gyrus	0.00157	Left	19
2	6,776	−48	−74	24	Middle Temporal Gyrus	0.007693	Left	39
2	6,776	−46	−72	36	Angular Gyrus	0.00742211	Left	39
3	5,960	−56	2	−28	Middle Temporal Gyrus	0.014750081	Left	21
3	5,960	−64	−20	−18	Middle Temporal Gyrus	0.010508	Left	21
3	5,960	−52	12	−12	Superior Temporal Gyrus	0.008636334	Left	22
3	5,960	−46	12	−18	Superior Temporal Gyrus	0.008573441	Left	38
3	5,960	−60	−10	−28	Inferior Temporal Gyrus	0.007971286	Left	20
**ALFF/fALFF**								
MCI>HC								
1	19,336	−6	−30	60	Paracentral Lobule	0.001944255	Left	5
2	14,864	−30	−78	−30	Tuber	0.006901412	Left	/
2	14,864	−18	−93	−15	Declive	0.006519429	Left	/
3	14,656	−42	−54	−15	Fusiform Gyrus	0	Left	37
4	13,168	16	−14	−22	Parahippocampal Gyrus	0.006627638	Right	34
4	13,168	−1	−2	−18	Hypothalamus	0.006446497	/	/
5	12,656	51	−60	−12	Fusiform Gyrus	0.007649368	Right	37
5	12,656	39	−69	−9	Declive	0.007187633	Right	/
**MCI < HC**								
1	27,400	−8	−48	38	Precuneus	0.008772571	Left	31
1	27,400	12	−60	34	Precuneus	0.007666224	Right	31
1	27,400	−6	−60	44	Precuneus	0.006712989	Left	7
1	27,400	14	−52	44	Precuneus	0.006358274	Right	7
2	13,736	−20	−10	−10	Medial Globus Pallidus	0.008748008	Left	/
2	13,736	−18	6	−4	Putamen	0.007649428	Left	/
**ReHo**								
MCI>HC								
1	688	−59	17	11	Inferior Frontal Gyrus	0.015190582	Left	44
2	560	56	14	14	Inferior Frontal Gyrus	0.016221888	Right	44
**MCI < HC**								
1	640	−58	−16	−6	Superior Temporal Gyrus	0.015689934	Left	21
2	640	−51	−42	45	Inferior Parietal Lobule	0.015689865	Left	40

*BA, Brodmann Area; ALE, Anatomical/Activation Likelihood Estimation; MNI, Montreal Neurologic Institute; MCI, amnestic mild cognitive impairment; HCs, healthy controls; ALFF, the amplitude of low frequency fluctuation; ReHo, regional homogeneity; FC, functional connectivity*.

#### Altered ReHo in MCI

**Figure 3:**
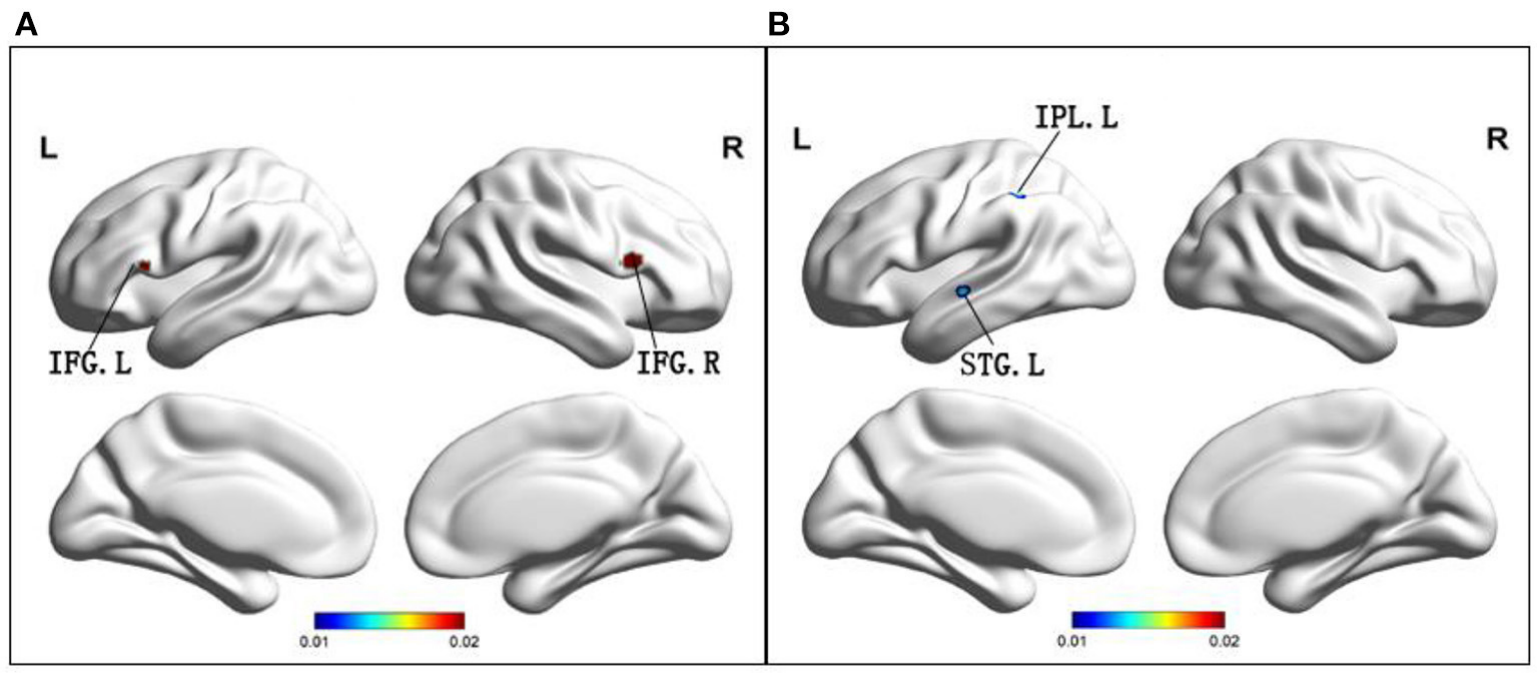
**(A)** Brain regions showing increased ReHo in MCI patients compared to HCs. **(B)** Brain regions showing decreased ReHo in MCI patients compared to HCs. MCI, amnestic mild cognitive impairment; ReHo, regional homogeneity; HCs, healthy controls; IFG, inferior frontal gyrus; IPL, inferior parietal lobule; STG, superior temporal gyrus; R, right; L, left.

Compared to HC, MCI patients demonstrated increased ReHo in the bilateral inferior frontal gyrus (Table 2 and Figure 3), while MCI patients showed decreased ReHo in the left superior temporal gyrus and left inferior parietal lobule (Table 2 and Figure 3).

#### Altered FC in MCI

**Figure 4:**
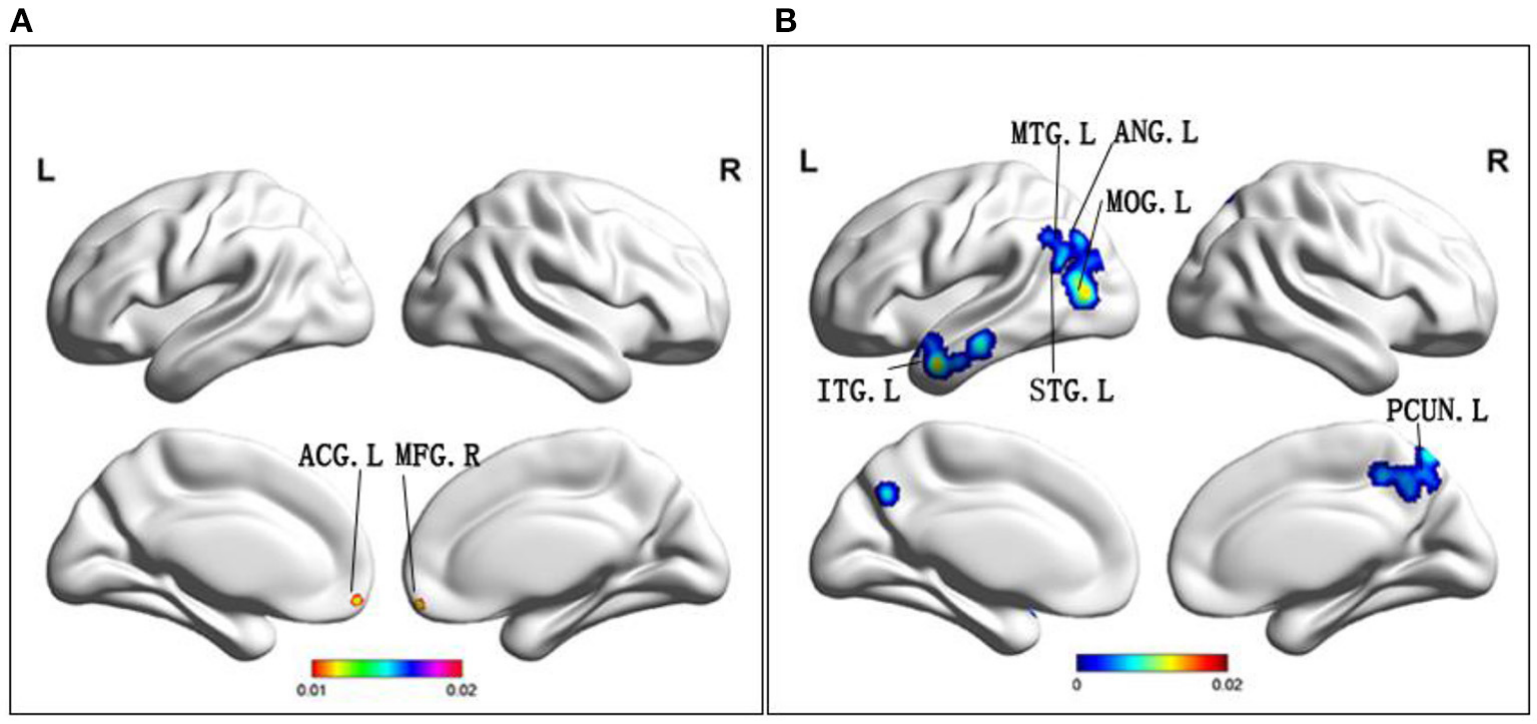
**(A)** Brain regions showing increased FC in MCI patients compared to HCs. **(B)** Brain regions showing decreased FC in MCI patients compared to HCs. MCI, amnestic mild cognitive impairment; FC, functional connectivity; HCs, healthy controls; ACG, anterior cingulate gyrus; MFG, middle frontal gyrus; ITG, inferior temporal gyrus; MTG, middle temporal gyrus; STG, superior temporal gyrus; ANG, angular gyrus; MOG, middle occipital gyrus; PCUN, precuneus; R, right; L, left.

Compared to HC, MCI patients demonstrated increased FC in the right medial frontal gyrus, left limbic lobe and left anterior cingulate cortex (ACC) (Table 2 and Figure 4). Conversely, MCI patients showed decreased FC in the bilateral precuneus, left middle occipital gyrus, right cingulate gyrus, left superior temporal gyrus, left middle temporal gyrus, left inferior temporal gyrus, left middle occipital gyrus, and left angular gyrus (Table 2 and Figure 4).

### Main Voxel-Wise Meta-Analysis

Among the different brain regions with FC changes, the precuneus was the largest, and the middle frontal gyrus and anterior angular gyrus were the smallest. The precuneus was shown to have largest changes in ALFF, and the smallest change occurred in the fusiform gyrus. Interestingly, the largest and smallest groups of changes in ReHo were in the inferior frontal gyrus, with a larger group on the left. The size of the point in the figure represents size of the cluster, and size of the cluster is consistent with its effect. This explanation applies to graphs with three different indicators.

## Discussion

In the past, there have been separate studies on three different indicators (ReHo, ALFF, FC) for DMN in MCI (Bai et al., 2008; Cai et al., 2015; Zhao et al., 2015). However, this was the first meta-analysis to conduct a comprehensive analysis of all three indicators. In our meta-analysis, the superior temporal gyrus, inferior temporal gyrus, parahippocampal gyrus, and precuneus were consistent with prior findings (Robinson et al., 2012). These results highlight the importance of altered DMN function in the pathophysiology of MCI. Furthermore, some of the altered brain regions have not yet been reported as belonging to DMN. Thus, perhaps the specific brain regions that are related to DMN remain to be explored. There is no denying that the brain regions that have specific changes may serve as biomarkers for diagnosis of MCI patients. Additionally, these brain regions can serve as biomarkers for predicting AD.

Firstly, it is known that changes in FC shown by patients with MCI are both complex and varied, depending on the study as well as method of use (Eyler et al., 2019). Previously, we found no evidence that changes in DMN connectivity were effective predictors of transition from MCI to AD (Xue et al., 2019). In our results, areas of FC increased mainly included the right medial frontal lobe and the left ACC. Some scholars propose that the increase of FC in the medial frontal lobe may be related to semantic memory deficiency (Su et al., 2017). However, maintenance of normal daily living functions during MCI may be the result of compensating for a disconnection after early hyperconnectivity of the medial frontal lobe (Wiepert et al., 2017). Another area of the brain that presents with increased FC was the left ACC. As is known, the PCC is the core area of DMN (Fransson and Marrelec, 2008). In AD, FC of PCC was severely damaged. In this study, the area where FC increased was ACC. On the other hand, previous studies have shown that the association between Theta activity in ACC and response time in the planning period may reflect the high cognitive demands associated with this task (Domic-Siede et al., 2021). Research supports that the anterior cingulate gyrus is well-suited to regulate behavioral selection and learning on multiple time scales, and to respond differently to environmental uncertainty and volatility (Monosov et al., 2020). Furthermore, increased ACC functional connectivity may be the result of making up for decreased PCC activity, thus maintaining relatively normal ability of daily living.

The increase or decrease of FC alone cannot be an effective diagnostic factor for the transition of MCI to AD. A combination of increase and decreased FC can improve sensitivity (Eyler et al., 2019). In our results, a decline in FC was mainly present in the bilateral precuneus and superior parietal lobes, both of which are responsible for visual, sensory, and motor integration. Previous studies have suggested that the precuneus is the “core node” of the DMN, and has been associated with high levels of amyloid deposition in early AD (Tao et al., 2017). As far as we know, deposition of protein and shrinkage of gray matter volume make DMN known to be a susceptible area of AD. However, whether early changes of FC in the precuneus, the core of DMN, can be regarded as a specific biomarker for the transition to AD, is unknown (Eyler et al., 2019). Another noteworthy area of FC decline is the middle temporal gyrus. The middle temporal gyrus (MTG) is understood to play a role in language-related tasks such as lexical comprehension and semantic cognition (Briggs et al., 2021). Furthermore, an increase of FC in the middle frontal gyrus may also be allowed to compensate for a decrease of FC in the medial temporal gyrus (Gardini et al., 2015). Previous literature has suggested that a FC decline during MCI and PCC-temporal cortex may be the central role of cognitive deficits in MCI patients (Fransson and Marrelec, 2008). However, the fact that the meta-analyses results are not clearly presented is also a limitation (Wiepert et al., 2017). In conclusion, changes in the FC of medial frontal gyrus, precuneus and middle temporal gyrus may be utilized as effective biomarkers to predict AD conversion.

The presence of ALFF reflects regional characteristics of brain activity. The study demonstrated that not all changed brain structures had a drop in ALFF, and there was no correlation between the changed brain and ALFF changes (Yin et al., 2014). One interesting result is that while the volume of gray matter in the frontal lobes, temporal lobes, and parietal lobes decrease, ALFF values can go up as well as down (Zou et al., 2015). Although results of ALFF remain controversial, their significance cannot be denied. The areas that demonstrate ALFF are noteworthy in the posterior lobe of the cerebellum, as well as the parahippocampal gyrus. The cerebellum is involved in cognitive, emotional, and sensory processing (Yin et al., 2014). In addition, a worthy increase in cerebellar ALFF may be a complement to cognitive deficits (Stoodley and Schmahmann, 2009; Wang et al., 2011). Parahippocampal gyrus and hippocampus located in the medial temporal lobe are the main brain regions that develop pathological change of AD. In addition, they play a crucial role in memory function, and may compensate for neurodegenerative changes following injury to MCI (Pan et al., 2017). Another area with a significant increase in ALFF is the fusiform gyrus. It is well-known that the fusiform gyrus is primarily involved in memory processing (Zhao et al., 2014). It has been found that the FC in the fusiform gyrus is also extensively altered when MCI patients perform facial-matching tasks (Xuan et al., 2012). The related memory and cognitive functions of MCI patients remain normal, which could not be ruled out as a compensation for increasing ALFF values in these specific regions.

A decrease of ALFF was mainly found in the bilateral precuneus. The precuneus acts as an intermediary between the semantic network, as well as the hippocampal memory system, encoding meaningful events into episodic memories (Schmahmann et al., 2007). Different from the ALFF decrease in the precuneus proposed in our study, most of the previous studies showed a decrease of FC in precuneus (Stoodley and Schmahmann, 2009; Wang et al., 2011). The exact mechanism, however, remains to be explored, which may enhance the credibility of the presence of abnormal neuroimaging markers in DMN during MCI. In conclusion, ALFF changes in the posterior cerebellar lobe, parahippocampal gyrus fusiform gyrus and precuneus may be utilized as predictors of AD conversion.

ReHo mainly explored differences of spontaneous activity in the whole brain. Both an increase and decrease in ReHo indicate changes in brain activity. With increase or decrease of ReHo value, regional metabolic rate constantly changes, and cerebral blood flow also increases or decreases (Bokde et al., 2006). The areas where ReHo dropped were mainly the superior temporal gyrus and the inferior parietal lobule. It is well-known that the parietal cortex has extensive connections with frontal lobes and controls sensory information for movement (Fransson and Marrelec, 2008). It has been shown that the left inferior parietal lobule is damaged among patients with MCI. Therefore, we hypothesize that a reduction of ReHo in the left inferior parietal lobule provides evidence for presence of DMN as a predictor of the conversion to AD.

Our meta-analysis demonstrated that the region where ReHo was elevated was the inferior frontal gyrus, which is one of the key regions of DMN (Huang et al., 2016), and is largely responsible for declarative long-term memory function (Sakurai and Gamo, 2019; Gao et al., 2020). Previous studies have demonstrated that the atrophy of the inferior frontal gyrus can predict conversion of MCI to AD (Yuan et al., 2013), so we may assume that elevation of ReHo value in the inferior frontal gyrus also has an important function in the conversion process of AD.

Finally, we discussed the brain regions that changed over the same time period and had overlapping indicators. The region of the brain where the FC and ALFF overlap was the precuneus. Both of the indexes were decreased in the precuneus. The areas where FC and ReHo overlap were the superior temporal gyrus, as well as the inferior parietal lobule. Interestingly, FC and ReHo decreased in both the superior temporal gyrus, and the inferior parietal lobule. It is well-known that impairment of the precuneus and inferior parietal lobule can cause memory impairment. The posterior superior temporal gyrus belongs to the Wernicke's area and may be associated with visuo-spatial function of MCI patients. Therefore, we suspect that there is a decrease in functioning of these regions, which may partially support occurrence of clinical symptoms.

In conclusion, a relationship between the three factors in predicting AD conversion remains to be considered. Although literature on neuroimaging studies of MCI is abundant, it is necessary to analyze and evaluate these studies. The significance of this meta-study is to try and identify consistent differential regions in DMN and provide specific biomarkers in a diagnosis of MCI. In this study, the middle frontal gyrus, cingulate gyrus, precuneus, temporal lobe, fusiform gyrus, parietal lobule and parahippocampal gyrus were utilized as biomarkers to predict the occurrence of AD. There is no doubt that our meta-analysis produced interesting and meaningful results, such as TMS therapy and drug therapy, which can be used to select the appropriate targets for optimal treatment. Our findings provide a specific imaging feature for diagnosis of MCI and is the basis for further research.

### Limitations

Although this meta-analysis has produced some interesting results, it has also some limitations. First of all, there is some heterogeneity in the subject's age, sex, years of education and other factors. However, none of these factors had a material effect on the outcomes. Next, we selected and included literature from different data sources, using different pre-processing, statistical and imaging methods, which led to some differences. In this case, we tried to select abundant data sources in the database in order to improve coverage of the literature, and select several researchers to identify the screening work during the extraction process. This further reduced difference in outcomes that resulted from this cause. Finally, we do not rule out the literature based on seed method, and selection of the seed is subject to the operator's subjective influence. The different seed selection point will influence results to some extent. Although seed-based algorithms are not excluded, inclusion of literature on such algorithms also enriched our results.

## Conclusion

Herein, we performed ALE meta-analysis in patients with MCI in order to determine functional changes in DMN. In our results, we found that function of altered brain regions were mainly in the precuneus, ACC, frontotemporal parietal lobe, some putamen and marginal lobe, among which the damage and compensatory mechanisms coexisted. Changes in these specific brain regions can help identify potential imaging biomarkers for MCI. It also explains the pathology of MCI, to a certain extent, and provides a novel specific target and direction for clinical diagnosis and treatment.

## Data Availability Statement

The original contributions presented in the study are included in the article/supplementary material, further inquiries can be directed to the corresponding author/s.

## Author Contributions

QY, WQ, CX, and JC designed the study. HG, GH, SC, WX, YS, and XZ screened the literature. QY, WQ, CX, and SC collected the data. QY and WQ: analyzed the data and prepared the manuscript. All authors contributed to the article and approved the submitted version.

## Funding

This study was supported by the National Natural Science Foundation of China (No. 81701675); the Key Project supported by Medical Science and technology development Foundation, Nanjing Department of Health (No. JQX18005); the Cooperative Research Project of Southeast University-Nanjing Medical University (No. 2018DN0031); the Key Research and Development Plan (Social Development) Project of Jiangsu Province (No. BE2018608); the Innovation and Entrepreneurship Training Program for College Students in Jiangsu Province (No.201810312061X; 201910312035Z); Jiangsu Provincial Natural Science Foundation-Youth Foundation Projects (BK20180370).

## Conflict of Interest

The authors declare that the research was conducted in the absence of any commercial or financial relationships that could be construed as a potential conflict of interest.

## Publisher's Note

All claims expressed in this article are solely those of the authors and do not necessarily represent those of their affiliated organizations, or those of the publisher, the editors and the reviewers. Any product that may be evaluated in this article, or claim that may be made by its manufacturer, is not guaranteed or endorsed by the publisher.
